# Glymphatic dysfunction evidenced by DTI-ALPS is related to obstructive sleep apnea intensity in newly diagnosed Parkinson’s disease

**DOI:** 10.1038/s41531-025-01018-8

**Published:** 2025-06-11

**Authors:** Jiri Nepozitek, Stanislav Marecek, Veronika Rottova, Simona Dostalova, Tomas Krajca, Jiri Keller, Karel Sonka, Petr Dusek

**Affiliations:** 1 Department of Neurology and Center of Clinical Neuroscience, First Faculty of Medicine, Charles University and General University Hospital in Prague, Prague, Czechia; 2 Faculty of Biomedical Engineering, Czech Technical University in Prague, Kladno, Czechia; 3 Radiodiagnostic Department, Na Homolce Hospital, Prague, Czechia; 4 Department of Radiology, First Faculty of Medicine, Charles University and General University Hospital in Prague, Prague, Czechia

**Keywords:** Parkinson's disease, Parkinson's disease, Parkinson's disease, Neurodegeneration, Risk factors

## Abstract

Glymphatic dysfunction potentially contributes to Parkinson’s disease (PD) via impaired clearance of metabolic waste products. Obstructive sleep apnea (OSA) can disturb sleep, which is necessary for proper glymphatic function, and is frequent in PD. We investigated the glymphatic function in de novo PD and its relation to OSA. Fifty-four PD patients (mean age 58.9 ± 12.2 years) and 32 controls (mean age 59.4 ± 8.3 years) underwent polysomnography and 3 T magnetic resonance imaging of the brain. Diffusion tensor imaging along the perivascular space (DTI-ALPS) was calculated using atlas-based automatic regions of interest selection. In PD, ALPS-index negatively correlated with apnea-hypopnea index (rho = −0.41; *p* = 0.002), oxygen desaturation index (rho = −0.38; *p* = 0.006), sleep stage N1 (rho = −0.42; *p* = 0.002), and arousal index (rho = −0.24; *p* = 0.018), while in controls, no such correlations were observed. Glymphatic dysfunction is related to OSA severity in de novo PD but not in controls. We suggest that OSA may contribute to neurodegeneration via glymphatic impairment in PD.

## Introduction

The glymphatic system is a highly organized fluid transport pathway subserving cerebrospinal fluid (CSF) flow via perivascular pathways^1,2^. Its main importance is in providing brain clearance for waste products, including neurotoxic protein aggregates implicated in the development of the neurodegenerative process^3–5^. Glymphatic system dysfunction has been documented to play a role in a variety of neurological conditions, such as Alzheimer’s disease, ischemic strokes, traumatic brain injury, normal pressure hydrocephalus, Parkinson’s disease (PD), and multiple sclerosis^6–13^.

The glymphatic system reaches its highest capacity during sleep^14–17^. Moreover, it has been proposed that the efficacy of glymphatic clearance relies on the depth of non-rapid-eye movement sleep (NREM), implying that the decrease of deep NREM sleep (NREM stage 3—N3), the frequent interruptions of lighter sleep stages (NREM stages 1 and 2 – N1 and 2), and the shorter total sleep time (TST), all worsen glymphatic activity^16–18^. Thus, several disorders and conditions are suspected of suppressing glymphatic function during NREM. Indeed, recently, it was documented that the glymphatic system is impaired in patients with obstructive sleep apnea (OSA)^19^.

In PD, the prevalence of OSA is repeatedly reported to be higher than in the general population^20^. The prevalence studies report that OSA is observed in 20–70% of PD patients. OSA comorbidity in PD has proven to exacerbate both motor and non-motor symptoms. Multiple pathogenetic relationships have been suggested in the interplay between OSA and PD, including intermittent hypoxemia, sleep fragmentation, upper airway obstruction, inflammation, and finally, alteration of the glymphatic system homeostasis^20^. Surprisingly, no studies have investigated the link between OSA and glymphatic dysfunction in PD to date.

Several compensatory mechanisms related to sleep and the glymphatic system are compromised in PD, contributing to disease progression^21^. PD is associated with disruptions in sleep architecture, including reduced N3, which contribute to glymphatic system impairment^22,23^. Reduced noradrenergic signaling resulting from degeneration of the locus coeruleus, a primary source of noradrenaline, could also contribute to diminished glymphatic function^24,25^. Dysfunction of aquaporin-4 (AQP4) in PD, which is crucial for glymphatic flow, hinders waste clearance^21,26^. Chronic neuroinflammation in PD can impair AQP4 function and glymphatic efficiency, reducing the brain’s ability to eliminate toxic proteins^21^. Collectively, these compromised mechanisms lead to the reduced clearance of neurotoxic substances, possibly contributing to the progression of PD.

Imaging of the glymphatic system and assessment of its function is an emerging field^1,2,27–30^. One of the suitable tools for its evaluation in humans is magnetic resonance imaging (MRI)^2^. Diffusion tensor imaging (DTI) along the perivascular space (DTI-ALPS) has been suggested as a method for the quantification of glymphatic system function^31,32^. It is based on evaluating the flow of water molecules in the direction of the perivascular spaces by measuring water diffusivity using DTI. This method relies on the assumption that the perivascular spaces along the medullary veins lie orthogonal to the projection and association fibers at the level of the lateral ventricle body^31,33^.

The DTI-ALPS technique involves placing regions of interest (ROIs) near the top of the lateral ventricles, specifically in areas containing projection and association fibers. Since glymphatic flow along venous perivascular spaces is perpendicular to the lateral ventricles in these regions, we can estimate glymphatic dysfunction by evaluating diffusivity in the three axes. The diffusivity in the x-axis is perpendicular to the ventricles and major tracts and contains the glymphatic flow. Conversely, the y- and z-axes for the projection and association areas, respectively, are perpendicular to both the major tracts and glymphatic flow. By comparing the diffusivity in these areas using the ALPS-index (Eq. 1), we can estimate glymphatic flow in the x-axis, excluding random diffusion values.

1 $$\mathrm{ALPS}-\mathrm{index}=\mathrm{mean}\,({{\rm{D}}}_{\mathrm{xx\; projection}},{{\rm{D}}}_{\mathrm{xx\; association}})/\mathrm{mean}\,({{\rm{D}}}_{\mathrm{yy\; projection}},{{\rm{D}}}_{\mathrm{zz\; association}})$$

In most studies that utilized the DTI-ALPS method, the ROI selection was performed manually. This introduces a possibility of human error and rater bias^34^. Several papers used an atlas-based automatic ROI selection method^35–37^, which does not share this limitation. To exclude undesirable voxels, some studies even utilized subsequent fractional anisotropy-based thresholding^38^.

This study aimed to use the DTI-ALPS method to evaluate glymphatic system function in patients with de novo PD to investigate its relationship to OSA severity and to parameters of sleep disruption and to compare these associations with healthy controls. We formulated the following hypotheses: (1) Glymphatic dysfunction is correlated with OSA intensity and parameters of sleep fragmentation. (2) Due to the possibility of early impairment of the compensatory mechanisms related to PD pathology that are maintained in healthy individuals, we expect these relationships to be more pronounced in PD patients compared to the control group.

## Results

### Characteristics of study participants

Compared to controls, PD patients had lower rapid-eye-movement sleep (R) stage ratios and lower arousal indexes. Other observed parameters, including age, ALPS index, apnea-hypopnea index (AHI), and oxygen desaturation index (ODI), did not show significant differences in the comparison analysis. Table 1 presents an overview of demographic and polysomnographic parameters and the ALPS-index.

**Table 1:** Comparison of demographic, clinical, and polysomnographic parameters and DTI-ALPS index

	PD (*N* = 54)		Controls (*N* = 32)			
	Mean	SD	Mean	SD	*p*	95% CI
Age (years)	58.9	12.2	59.4	8.3	0.907	0.906–0.917
Gender (F/M)	17/37		10/22		1.000	
Body Mass Index	27.5	3.7	27.6	4.0	0.908	0.906–0.917
MDS UPDRS III	30.2	14.0				
ALPS-Index	1.29	0.164	1.34	0.156	0.093	0.091–0.102
Total Sleep Time (min.)	334.4	255.2	332.1	72.3	0.235	0.534–0.554
Sleep Latency (min.)	25.9	37.3	13.9	13.0	0.190	0.404–0.423
Sleep Efficiency (%)	66.8	21.5	74.9	14.6	0.122	0.344–0.363
Wake (%)	30.0	20.4	22.7	13.8	0.160	0.439–0.459
Stage N1 (%)	7.9	5.1	8.6	4.0	0.129	0.065–0.075
Stage N2 (%)	33.1	12.6	35.3	9.9	0.457	0.450–0.469
Stage N3 (%)	16.7	9.8	17.4	7.1	0.621	0.889–0.901
Stage R (%)	12.2	7.9	16.0	7.0	0.021^a^	0.253–0.271
Apnea-Hypopnea Index	16.0	19.9	18.5	15.8	0.076	0.073–0.083
Oxygen Desaturation Index	14.2	17.1	13.9	11.4	0.282	0.333–0.351
Periodic Limb Movements Index	8.9	19.5	10.6	23.0	0.590	0.620–0.639
Arousal Index	8.1	7.2	16.6	7.5	**<0.0001**^**a**^	0.000–0.000

Results are presented as mean and standard deviation. Mann-Whitney *U* test was applied. Bold: Significant after Bonferroni correction.

*F* females, *M* males, *MDS-UPDRS* Unified Parkinson’s Disease Rating Scale, *ALPS* analysis along the perivascular space, *N1, 2, 3* non-rapid eye movement sleep stage 1, 2, 3, *R* rapid eye movement sleep, *PD* Parkinson’s disease, *N* number, *SD* standard deviation, *CI* confidence interval.

^a^Difference is significant at the 0.05 level.

### Associations of DTI-ALPS with observed parameters

**Fig. 1:**
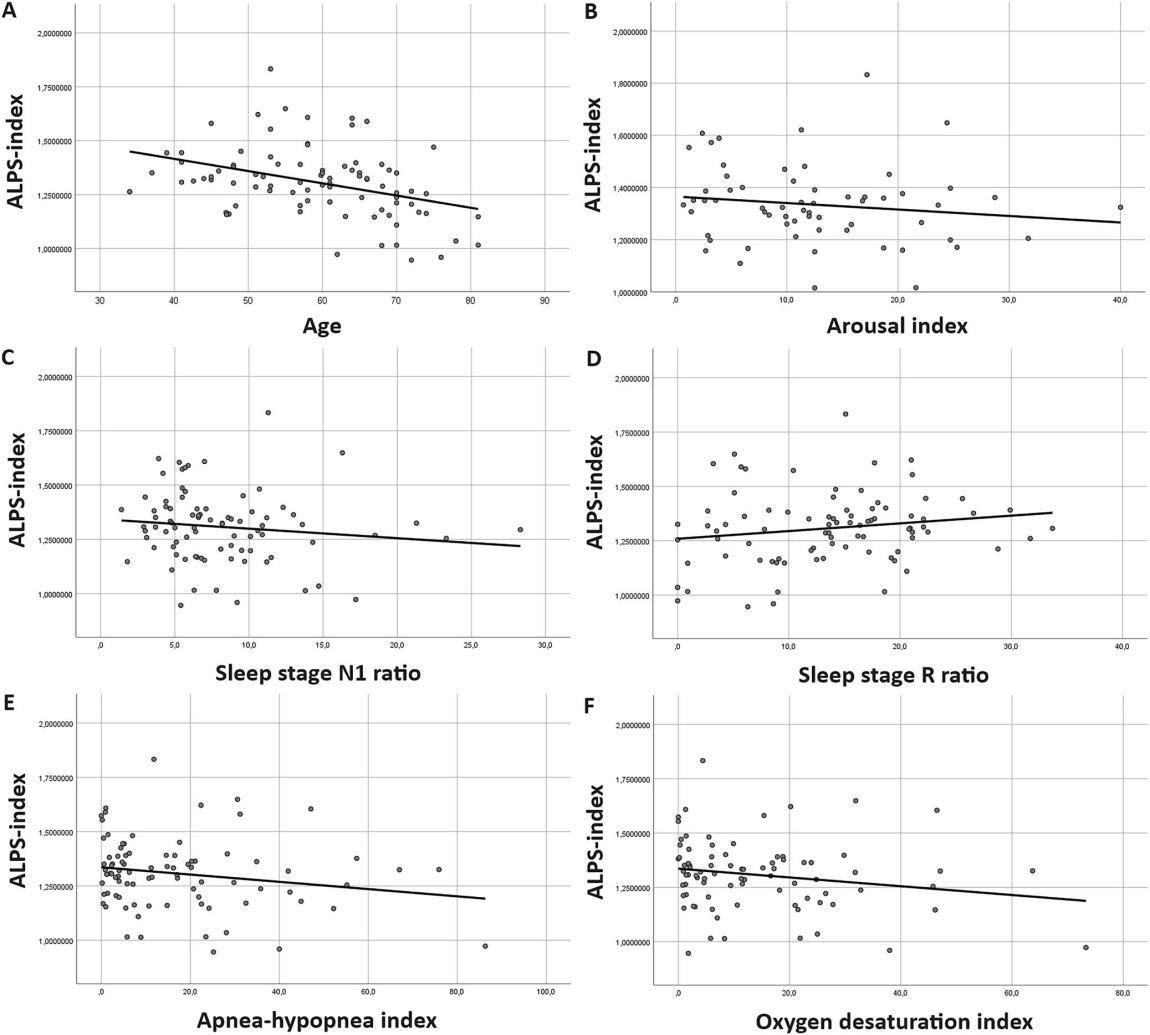
Correlation plots showing relation of ALPS-index to observed parameters in PD patients. Correlation analysis between ALPS-index and age (**A**), arousal index (**B**), sleep stage N1 ratio (**C**), sleep stage R ratio (**D**), apnea-hypopnea index (**E**), and oxygen desaturation index (**F**) in Parkinson’s disease. Legend: ALPS analysis along the perivascular space, N1 non-rapid eye movement sleep stage 1, R rapid eye movement sleep.

The ALPS-index was negatively correlated with age in both groups. In the PD group, the ALPS-index was significantly negatively correlated with AHI, ODI, sleep stage N1 ratio, and arousal index, and significantly positively correlated with sleep stage R ratio (Fig. 1). This contrasted with the control group, where the ALPS-index was only correlated with age (Table 2).

**Table 2:** Correlations between ALPS-index and observed parameters

Correlations	PD			Controls		
ALPS-index vs.:	rho	*p*	95% CI	rho	*p*	95% CI
Age	−0.36^a^	0.007	−0.547 to 0.268	−0.56^a^	**0.001**	−0.747 to −0.279
Body Mass Index	−0.12	0.388	−0.299 to 0.521	0.25	0.167	−0.180 to 0.541
MDS UPDRS III	−0.24	0.085	−0.620 to 0.139	−0.08	0.672	−0.503 to 0.304
Total Sleep Time	0.04	0.785	−0.325 to 0.602	0.00	0.99	−0.283 to 0.390
Sleep Latency	0.03	0.854	−0.382 to 0.511	0.16	0.385	−0.235 to 0.432
Sleep Efficiency	0.19	0.184	−0.283 to 0.628	0.13	0.495	−0.167 to 0.532
Wake ratio (%)	−0.20	0.153	−0.661 to 0.245	−0.18	0.335	−0.595 to 0.134
Stage N1 ratio (%)	−0.42^a^	**0.002**	−0.589 to 0.099	0.21	0.239	−0.12 to 0.580
Stage N2 ratio (%)	0.11	0.419	−0.290 to 0.573	0.14	0.461	−0.247 to 0.583
Stage N3 ratio (%)	0.11	0.423	−0.245 to 0.637	−0.03	0.885	−0.406 to 0.369
Stage R ratio (%)	0.31^b^	0.023	−0.259 to 0.629	−0.13	0.49	−0.39 to 0.278
Apnea-Hypopnea Index	−0.41^a^	**0.002**	−0.718 to 0.215	0.09	0.615	−0.322 to 0.409
Oxygen Desaturation Index	−0.38^a^	**0.006**	−0.689 to 0.174	0.03	0.864	−0.368 to 0.393
Periodic Limb Movements Index	−0.21	0.144	−0.684 to 0.062	−0.28	0.123	−0.587 to 0.151
Arousal Index	−0.42^b^	0.018	−0.794 to −0.133	0.10	0.606	−0.294 to 0.451

Bold: Significant after Bonferroni correction.

*ALPS* analysis along the perivascular space, *MDS-UPDRS* Unified Parkinson’s Disease Rating Scale, *N1, 2, 3* non-rapid eye movement sleep stage 1, 2, 3, *R* rapid eye movement sleep, *PD* Parkinson’s disease, *CI* confidence interval.

^a^Correlation is significant at the 0.01 level (2-tailed).

^b^Correlation is significant at the 0.05 level (2-tailed).

The relationship of glymphatic function to gender was investigated using a comparison of ALPS-indices between females and males. In the PD group, males showed lower ALPS-indices compared to females (1.254 ± 0.163 vs.1.355 ± 0.148; *p* = 0.040), while in the control group, no differences in ALPS-indices were found when comparing gender.

Among other relevant parameters in PD, AHI and ODI correlated with sleep efficiency (SE) (AHI: rho = −0.34; *p* = 0.012, ODI: rho = −0.34; *p* = 0.015), with wakefulness ratio (AHI: rho = 0.37; *p* = 0.007; ODI: rho = 0.37; *p* = 0.008), with sleep stage N1 ratio (AHI: rho = 0.31; *p* = 0.025, ODI: rho = −0.41; *p* = 0.003), with sleep stage N3 ratio (AHI: rho = −0.36; *p* = 0.008; ODI: rho = −0.34; *p* = 0.017), with sleep stage R (AHI: rho = −0.53; *p* < 0.0001, ODI: rho = −0.49; *p* < 0.0001). While the arousal index was correlated with ODI (rho = 0.40; *p* = 0.036), we could not find a correlation with AHI.

To evaluate the effect of the relevant parameters on the main results, we applied multivariable regression with ALPS-index as the dependent variable and explanatory parameters: AHI, age, gender, SE, periodic limb movements index (PLMI), group, and interaction term between AHI and group. This regression showed a significant effect of age and gender, and we also observed a significant interaction indicating that the relationship between the ALPS-index and AHI differs depending on the group (Table 3). As a result of that, we conducted a post-hoc multivariable regression analysis in the PD and control groups separately. In PD, we found significant effects of age, gender, and AHI on the ALPS-index, while in controls, we found only the significant effect of age (Table 4).

**Table 3:** Multivariable regression analysis with interaction term between AHI and group

Dependent Variable: ALPS-index	*B*	Std. Error	Beta	*t*	*p*
(Constant)	1.824	0.138		13.184	0.000
AHI	0.002	0.002	0.178	0.957	0.342
Age	−0.005	0.002	−0.354	−3.516	0.001
Gender	−0.116	0.034	−0.327	−3.423	0.001
Sleep Efficiency	−0.001	0.001	−0.152	−1.293	0.200
PLMI	−0.001	0.001	−0.139	−1.419	0.160
Group	−0.006	0.047	−0.017	−0.125	0.901
Interaction Term: AHI−Group	−0.004	0.002	−0.446	−2.154	0.034

Multivariable regression was applied with ALPS-index as a dependent variable and explanatory parameters including interaction term between AHI and group.

*ALPS* analysis along the perivascular space, *AHI* apnea-hypopnea index, *PLMI* periodic limb movements index.

**Table 4:** Post-hoc multivariable regression analysis assessing the effect of covariates within the separate groups: PD patients and controls

Dependent Variable: ALPS-index	*B*	Std. Error	Beta	*t*	*p*
PD patients					
(Constant)	1.811	0.168		10.757	0.000
AHI	−0.003	0.001	−0.349	−2.158	0.036
Age	−0.005	0.002	−0.347	−2.570	0.014
Gender	−0.123	0.046	−0.345	−2.674	0.010
Sleep Efficiency	−0.002	0.001	−0.206	−1.195	0.238
PLMI	−0.001	0.001	−0.131	−0.917	0.364
Controls					
(Constant)	1.900	0.271		7.018	0.000
AHI	0.001	0.002	0.148	0.876	0.389
Age	−0.008	0.003	−0.421	−2.457	0.021
Gender	−0.083	0.055	−0.252	−1.509	0.143
Sleep Efficiency	−0.001	0.002	−0.054	−0.316	0.755
PLMI	−0.001	0.001	−0.195	−1.163	0.256

Multivariable regression was applied with ALPS-index as a dependent variable and explanatory parameters.

*ALPS* analysis along the perivascular space, *PD* Parkinson’s disease, *AHI* apnea-hypopnea index, *PLMI* periodic limb movements index.

### Sleep comorbidities

Rapid-eye-movement sleep behavior disorder (RBD) was detected in 8 PD patients (14.8%), the restless legs syndrome (RLS) in 1 patient (1.9%), and periodic limb movements in sleep (PLMS) in 10 patients (18.5%).

Sleep comorbidities were evaluated as potential confounders using multivariable regression. After adjustment for RBD, RLS, and PLMS, the ALPS-index stayed significantly associated with AHI (Table 5).

**Table 5:** Effect of comorbidities as potential confounders to association between ALPS-index and AHI in PD

Dependent Variable: ALPS-index	*B*	Std. Error	Beta	*t*	*p*
(Constant)	1.366	0.030		45.172	0.000
AHI	−0.003	0.001	−0.376	−2.898	0.006
RBD	−0.111	0.059	−0.241	−1.865	0.068
RLS	−0.131	0.162	−0.108	−0.808	0.423
PLMS	−0.066	0.056	−0.159	−1.190	0.240

Multivariable regression was applied with ALPS-index as a dependent variable and explanatory parameters.

*AHI* apnea-hypopnea index, *ALPS* analysis along the perivascular space, *RBD* rapid-eye-movement sleep behavior disorder, *RLS* restless legs syndrome, *PLMS* periodic limb movements in sleep.

## Discussion

This study is the first to elucidate previously unexplored associations of glymphatic dysfunction in de novo PD with OSA parameters and other polysomnographic features. The major finding of this study was that in de novo PD patients, glymphatic system function was related to several parameters, particularly polysomnographic, which were absent in controls. The glymphatic function was negatively correlated with AHI, ODI, and sleep stage N1 ratio.

The ALPS-index, suggesting dysfunction of the glymphatic system, is presumably associated with the process of neurodegeneration and with neurodegenerative diseases, including PD^10,21^. In our study, glymphatic system dysfunction was related to OSA severity in PD patients. This finding is consistent with a recent report performed on a cohort of individuals with severe OSA^19^. OSA is frequent in PD, and it exacerbates both motor and non-motor symptoms of PD^20^. The influence of comorbid OSA on PD through dysfunction of the glymphatic system has only been hypothesized without direct evidence. This study documents the missing link between OSA and glymphatic system dysfunction in PD. PD patients in our study showed no difference in OSA intensity compared to controls of equal age. Our findings indicate that OSA might have a more pronounced impact on patients with PD already at the time of diagnosis. While one possible explanation is that the neurodegenerative process in PD increases the brain’s vulnerability to OSA and its consequences, causality cannot be established due to the cross-sectional design of our study. Therefore, alternative interpretations should also be considered, including the possibility that glymphatic dysfunction and OSA represent parallel manifestations of PD without a direct causal link.

Considering that the glymphatic system is most active in sleep, particularly dependent on TST and deep sleep stages (N3) amounts, fragmented sleep is regarded as a pathogenetic mechanism that leads to glymphatic dysfunction^16–18^. In this study, in PD, we found a negative association of glymphatic function with the frequency of arousals, which is a direct parameter of sleep fragmentation, and further with the sleep stage N1 ratio, the shallowest sleep stage. We also consider this to be a manifestation of sleep disruption, as N1 is the initial sleep stage following a sleep interruption manifested as arousal, and its ratio increases proportionally when the ratio of deeper sleep stages decreases. In accordance with this, we found a positive association of glymphatic function with the proportion of sleep stage R, the normal proportion of which is conditioned by the complete uninterrupted course of the entire sleep cycle, so it can be considered a manifestation of maintained sleep continuity. Therefore, our results are in line with the assumption that glymphatic dysfunction has a relationship with sleep fragmentation in PD. It has been implied that the relationship between sleep fragmentation, glymphatic dysfunction, and the process of neurodegeneration is linked in potential bidirectional relationships that could accelerate pathophysiological processes over time^13,21^. Our results show that this “vicious triangle” starts already at the time of PD diagnosis; it can indeed be expected that the condition will progress during the PD progression and that the relationships between the mentioned parameters will be stronger in more advanced stages of the disease.

Causal relationships between fragmented sleep and OSA are bidirectional. Sleep fragmentation is a known consequence of OSA; on the other hand, a low arousal threshold and associated sleep fragmentation are well-recognized contributors to OSA pathophysiology in specific phenotypes of the disorder^39^. In our study, most parameters that correlated with glymphatic dysfunction were also associated with OSA, as indicated by their correlation with AHI and ODI. In particular, these included decreased SE, an increase in sleep stage N1 ratio, and a decrease of sleep stage R ratio. The finding that the arousal index was not related to AHI, although it correlated with DTI-ALPS, suggests that other mechanisms could be involved. Sleep fragmentation is well documented in PD^40^ and has multiple causes beyond OSA, including muscle cramps, periodic limb movements, increased muscle tension, and pathophysiology of the disease itself^41^. However, the arousal index was related to ODI, which points to the hypoxic events and, therefore, still supports OSA as the possible mediator.

Besides sleep fragmentation, OSA leads to recurrent hypoxia. Hypoxia was suggested as a likely mechanism through which OSA increases the risk for cognitive impairment^42^. It was demonstrated that OSA is associated with declines in memory, attention, and executive functions in adults^43^. Further, it was shown that glymphatic system dysfunction is correlated with ODI^19^, which is consistent with our findings demonstrating the same relationship. We conclude that OSA enters the “vicious triangle” pathogenesis with worsening sleep disruption and hypoxic burden. Therefore, we stress that OSA should be actively screened for at the moment of PD diagnosis.

Surprisingly, the relationship of glymphatic dysfunction to OSA and other sleep parameters observed in PD was not expressed in the control group. The mere presence of early PD unmasked these relationships, which were apparently not present in the control group. It can be concluded that the neurodegenerative process creates a pathogenetic substrate of vulnerable brain tissue for the pathophysiological relationships and events described above. At this point, it is appropriate to emphasize that control subjects did not differ in the intensity of OSA or the proportions of NREM sleep stages, and the frequency of arousals was even higher in the control group. It seems that in control subjects, there could be a compensatory mechanism that prevents the development of these relationships observed in de novo PD.

Although the connection between OSA and glymphatic function was previously reported in a general OSA sample^19^, we did not find the same association in our study’s control group. The reason is probably that in the study by Lee et al., a group with diagnosed OSA was purposefully selected, whereas in our study, the control group was a general population sample. The selection-induced difference in the prevalence and severity of OSA between the groups here probably caused the loosening of the link with glymphatic function in our study’s group of healthy controls.

Our results on the relation of OSA to glymphatic impairment in PD are consistent with a very recent study reporting the relation of glymphatic dysfunction to body mass index (BMI) in PD^44^. OSA is known to be closely associated with obesity^45^. Therefore, it is reasonable to consider that OSA may have contributed to the mediation of the main finding of the mentioned study. Surprisingly, in our study, the ALPS-index did not correlate with BMI, and BMI was not significantly different between PD patients and controls.

In our study, we did not find a significant glymphatic dysfunction in PD patients compared to controls; however, the apparent trend to lower ALPS-index values in the PD group is in agreement with a recent study^46^. Unlike previous studies investigating glymphatic function in PD, PD patients were at the very threshold of diagnosis in our study. That could be why their glymphatic function impairment may not have been so pronounced. This could also be due to a relatively low number of subjects in our cohorts, which may have led to limited statistical power.

The negative correlation of glymphatic function with age in both groups is consistent with the available literature^3,47–49^. The gradual decrease of glymphatic function during the aging of the brain is apparently a parallel process, most likely a result of changes in cerebrovascular function with age that lie in the background of the events discussed above. Our control group’s results show that it does not avoid even healthy individuals.

There are limitations to this study that should be noted. The gender comparison in our study may have been underpowered due to the sample size distribution, which limits the ability to detect potential differences reliably. Future studies with larger and more balanced samples are needed to confirm the findings.

It also needs to be stated that while the DTI-ALPS method provides valuable insights into glymphatic system function and is frequently used in contemporary studies, its limitations have recently been pointed out. One of the main critiques of the DTI-ALPS method is that it includes the ability to capture only a specific aspect of its glymphatic dynamics, which may not fully represent the overall state of the glymphatic system. Therefore, the ALPS-index may not directly equate to glymphatic system function^32^. However, our study employed an enhanced approach incorporating automatic atlas-based analysis, improving precision and mitigating the method’s limitations.

We demonstrated that glymphatic system dysfunction is related to the severity of OSA and parameters of sleep fragmentation in newly diagnosed PD but not in controls. Thus, we documented for the first time the link between OSA and glymphatic system dysfunction in PD. It might be suggested that OSA contributes to the neurodegenerative process via glymphatic impairment in PD. Therefore, we recommend that OSA should be actively screened for at the moment of PD diagnosis.

## Methods

### Study participants

Fifty-four treatment-naive PD patients newly diagnosed at our Movement Disorders Center at the Department of Neurology and Center of Clinical Neuroscience, First Faculty of Medicine, Charles University, and General University Hospital in Prague were consecutively included in the study. The diagnosis was confirmed by a movement disorders specialist (P.D.) according to the Movement Disorders Society (MDS) clinical diagnostic criteria^50^. The exclusion criteria were treatment with antiparkinsonian medication before baseline examination, clinical, imaging, or laboratory signs of atypical parkinsonism, and normal findings on dopamine transporter single-photon emission computed tomography (DAT-SPECT) examination^51^. Thirty-two control subjects were recruited from the general community through advertisements. To be eligible for the study, controls had to be free of major neurologic disorders, active oncologic illness, and abuse of psychoactive substances.

All study participants underwent a protocol consisting of a comprehensive medical history, a neurological examination including the Unified Parkinson’s Disease Rating Scale (MDS-UPDRS), polysomnography, and brain MRI.

The study was approved by the Ethics Committee of the General University Hospital in Prague, and participants signed written informed consent before entering the study in accordance with the Helsinki Declaration.

### Polysomnography

Nocturnal polysomnography was performed using a digital polysomnography system (RemLogic, version 3.4.1, Embla Systems). It consisted of electrooculography, electroencephalography (F3-M2, C3-M2, O1-M2, F4-M1, C4-M1, O2-M1), surface electromyography (EMG) of the bilateral mentalis and tibialis anterior muscles, electrocardiography, nasal pressure, nasal and oral airflow, thoracic and abdominal respiratory effort, oxygen saturation, microphone, and digitally synchronized video monitoring, measured during the period from 10 p.m. to 6 a.m. according to the American Academy of Sleep Medicine (AASM) recommendation^52^. All features on PSG were analyzed visually. The sleep stages, respiratory events, limb movements, and arousals were scored according to the AASM Manual for the Scoring of Sleep and Associated Events version 2.2 2015^52^. Thus, we obtained the following sleep characteristics: TST, sleep latency (SL), SE, the ratio of wakefulness referring to the percentage of total recording time, sleep stage N1, N2, N3, and R, arousal index, AHI, ODI, and PLMI.

The presence of comorbid RBD was detected in PD based on behavioral events during sleep stage R on the video and prominent EMG activity during sleep stage R demonstrated at the nocturnal polysomnography^53^. Comorbid RLS in PD was diagnosed clinically according to the International RLS Study Group criteria^54^. The diagnosis of comorbid PLMS in PD was based on the AASM criteria (ICSD-3), which requires PLMI > 15^53^.

### Imaging acquisition protocol

MRI examination was carried out on a 3T scanner (Siemens Skyra, Siemens Healthcare, Erlangen, Germany) with a 32-channel head coil. The protocol included (1) a diffusion tensor MRI with repetition time (TR) = 10.5 s; echo time (TE) = 93 ms; total 72 slices with voxel resolution of 2 mm isotropic; 30 noncolinear directions with *b* value of 1000 s/m^2^, one b = 0 s/m^2^ image in antero-posterior and one *b* = 0 s/m^2^ image in postero-anterior phase encoding direction, (2) an axial 3D T1-weighted Magnetization Prepared Rapid Gradient Echo (MPRAGE, TR 2200 ms; TE 2.4 ms; inversion time (TI) 900 ms; flip angle (FA) 8°; field of view (FOV) 230 × 197 × 176 mm; voxel resolution 1 × 1 × 1 mm^3^).

### DTI-ALPS calculation

We measured the mean ALPS index of both hemispheres using an automatic atlas-based approach with subsequent color-coded fractional anisotropy thresholding. We chose this approach to avoid ROI selection bias and developed a color-coded fractional anisotropy thresholding to exclude voxels with a low inclination towards the desired axes. The analysis was performed in each subject’s native diffusion space, and the DWI data were corrected for distortion, movement artifacts, and eddy currents using FSL’s *topup* and *eddy* tools.

**Fig. 2:**
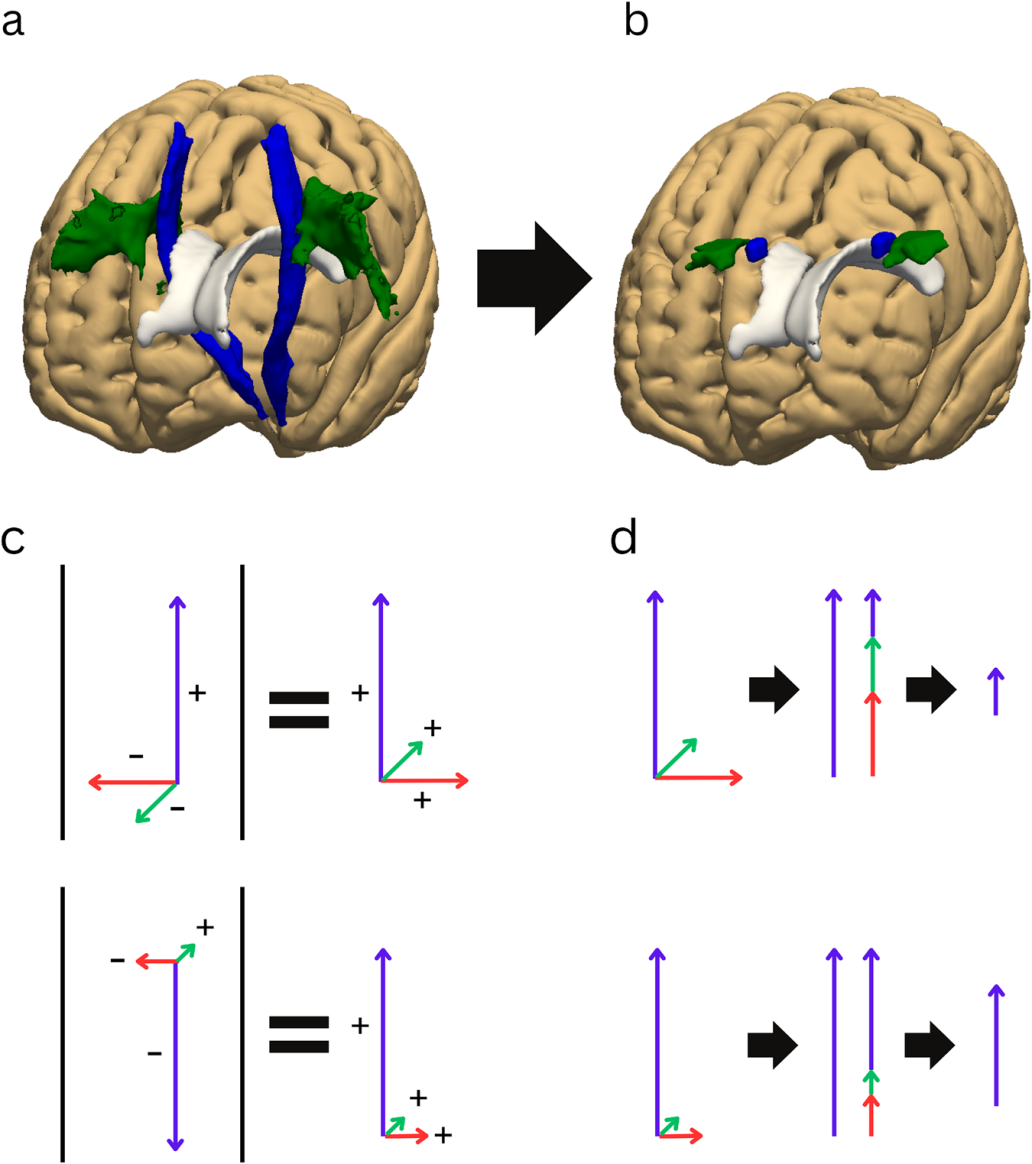
Preprocessing steps of the automatic approach. Using atlas regions of interest (**a**), restricting to the desired areas (**b**). Then, using color-coded fractional anisotropy thresholding by acquiring absolute values of the principal eigenvector (**c**) and subtracting the nuisance vectors from the desired one (**d**) and multiplying the result by fractional anisotropy.

We used the “JHU ICBM tracts maxprob thr25 1 mm” atlas’s labels of superior longitudinal fasciculus (SLF) and corticospinal tract (CST) for the association and projection areas, respectively^55^ (Fig. 2a). We restricted these labels to areas at the top of the lateral ventricles and areas with a low probability of intruding into the cortex during transformations to each subject’s diffusion space with the resulting masks (Fig. 2b). These restricted labels were then used as precursors to the final ROIs and were transformed from the MNI152 space to each subject’s native diffusion space. To achieve these transformations, each T1 image was first transformed into MNI152 space using FSL’s *flirt* and *fnirt* tools^56^. Subsequently, the original T1 image was coregistered to its corresponding diffusion image via *flirt*. The *convertwarp* function was then employed to generate a single transformation from the MNI152 space to the subject’s native diffusion space. Each transformation was manually inspected for errors.

Using FSL’s *dtifit*, we acquired each voxel’s principal eigenvector, defined by three volumes representing the x/y/z axes, and transformed these into absolute values (Fig. 2c). As a result, we obtained three volumes representing the principal eigenvector’s inclination to each of the x/y/z/ axes, respectively, but we lost the precise direction of the principal eigenvector.

We analyzed the ROIs for each hemisphere separately. For the projection ROIs, we subtracted the x- and y-volumes from the z-volume. The resulting image favored the “purest” diffusion in the z-axis (Fig. 2d). An analogous approach was used to obtain the association area ROIs. The 87% highest-value voxels restricted to the CST and SLF masks were used as the projection and association area ROIs, respectively.

Subsequently, we acquired the diffusivity images in the x/y/z axes using FSL’s *dtifit*. The diffusivity values used in the ALPS-index (Eq. 1) were then extracted from these images using the projection and association area ROIs acquired in the previous steps. The average ALPS-index of both hemispheres was calculated for each subject. All resource-heavy computation steps were performed on the MetaCentrum distributed computing infrastructure.

### Statistical analysis

The Shapiro-Wilk test was used to test the normality of the data distribution. Comparisons were conducted using the Mann–Whitney *U* test. Spearman’s rank correlation coefficient (rho) was used for the correlation analysis. Statistical significance was defined as a two-tailed *p*-value less than 0.05. The Bonferroni method was applied to correct familywise error to all comparison and correlation analyses. We conducted a multivariable regression analysis including an interaction term to examine whether the relationship between ALPS-index and AHI differs depending on the group. The multivariable regression was then used as a post-hoc analysis to evaluate the effect of explanatory parameters in the groups separately. All statistical analyses were performed using SPSS Software (IBM SPSS Statistics Version 26).

## Data Availability

The datasets used and/or analyzed during the current study are available from the corresponding author on request.
